# Identification of paternal germline mosaicism by MicroSeq and targeted next‐generation sequencing

**DOI:** 10.1002/mgg3.1394

**Published:** 2020-07-09

**Authors:** Congling Dai, Dehua Cheng, Weina Li, Sicong Zeng, Guangxiu Lu, Qianjun Zhang

**Affiliations:** ^1^ Institute of Reproductive and Stem Cell Engineering School of Basic Medical Science Central South University Hunan China; ^2^ Reproductive and Genetic Hospital ofCITIC‐Xiangya Hunan China; ^3^ School of medicine Hunan Normal University Hunan China; ^4^ Hunan Guangxiu Hospital Hunan China

**Keywords:** chromosome microdissection, *COL4A3*, germline mosaicism, targeted next‐generation sequencing

## Abstract

**Background:**

Prezygotic de novo mutations may be inherited from parents with germline mosaicism and are often overlooked when the resulting phenotype affects only one child. We aimed to identify paternal germline mosaicism in an index family and provide a strategy to determine germline mosaicism.‘

**Methods:**

Whole‐exome sequencing was performed on an Alport syndrome‐affected child. Variants were validated using Sanger sequencing in the pedigree analysis. An apparent de novo mutation was tested by next‐generation sequencing (NGS) following chromosome microdissection of the mutant region (MicroSeq) to clarify its homologous chromosome source. Mosaic mutation in sperm samples was detected using targeted next‐generation sequencing (TNGS). Self‐prepared mosaic DNA samples of the 3% and 0.1% mutant fractions were used to evaluate the TNGS detection sensitivity.

**Results:**

Two novel heterozygous variants, maternally inherited c.1322delT (p.Ile441Thrfs*17) and the de novo mutation c.2939T>A (p.Leu980Ter), in the *COL4A3* gene were discovered in the propositus. MicroSeq identified c.2939T>A in the paternal chromosome, which was *in trans* with c.1322delT. The frequency of c.2937A was 2.65% in the father's sperm sample. We also showed that a 500X depth coverage may detect a mosaic mutation with an allele frequency as low as 2%–3% using TNGS.

**Conclusion:**

MicroSeq is a valuable tool to identify the allele source of de novo mutations in a single patient. TNGS can be used to assess the mosaic ratios of known sites. We provided a systematic algorithm to detect germinal mosaicism in a single patient. This algorithm may have implications for genetic and reproductive counseling on germline mosaicism.

## Graphical abstract

we reported for the first time the application of MicroSeq in the diagnosis of Mendelian hereditary disease, which was a technique combined chromosome microdissection and next‐generation sequencing (NGS). We used targeted next‐generation sequencing (TNGS) to assess the mosaic ratios of known site and found that 500X reads depths coverage could meet the detection requirements of low‐frequency mosaic mutation of 2% to 3%.

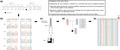

## 1 INTRODUCTION

Mutations detected in the propositus but absent from the parents are usually perceived as a de novo mutation in routine genetic testing using blood samples, but in fact, many apparently de novo mutations in the patient might be a consequence of somatic or germline mosaicism in unaffected parents (Campbell et al., 2014). Many apparently de novo mutations have been reported to be inherited from a parent with germline mosaicism and affect several Mendelian diseases, such as campomelic dysplasia (Higeta et al., 2018), fragile X syndrome (Jiraanont et al., 2016), and branchio‐oto syndrome (Miyagawa, Nishio, Hattori, Takumi, & Usami, 2015), and even accounted for 14.6% (6/41) of Duchenne muscular dystrophy cases (Bakker et al., 1989). The omission of germline mosaicism may lead to the recurrence of diseases and bring extra emotional and economic burdens to the affected family.

Currently, a series of problems have complicated identification of mosaicism in germ cell compartments. First, when two or more affected children are born to apparently unaffected parents, germline mosaicism is suspected; however, in first‐generation offspring, this event will usually be neglected when only one offspring has been affected (Mohrenweiser & Zingg, 1995).

Second, haplotype analysis using microsatellite markers surrounding the target gene is traditionally used to distinguish the paternal and maternal alleles and infer germinal mosaicism when there is more than one affected or unaffected siblings (Anazi, Al‐Sabban, & Alkuraya, 2014); this method cannot provide direct evidence of germline mosaicism and may be uninformative when there is only a single‐affected offspring and unaffected offspring. Therefore, a practical method is needed under this situation. As a bridge between cytogenetics and molecular genetics, chromosome microdissection (CM) was first developed by Scalenghe et al in 1981 (Scalenghe, Turco, Ederström, Pirrotta, & Melli, 1981) and has led to a number of applications: genetic linkage map, physical map construction, and expressed sequence tag generation (Arens et al., 2004). In recent years, CM was used for identification of the intertranslocation and breakpoint of the chromosome when combined with DNA sequencing, which was called MicroSeq (Hu et al., 2016). Theoretically, we believe that CM has the potential to haplotype a single individual. However, to date, there have been no reports on this application.

Third, when mutations come from the maternal chromosome, maternal germline mosaicism cannot be identified because eggs are not easily obtained. Regarding paternal germline mosaicism, sequencing sperm DNA is an approach to distinguish germline mosaicism. Several methods are implemented in mosaic mutation sequencing, including Sanger sequencing, high‐resolution melting (HRM) analysis, allele‐specific PCR, pyrosequencing, SNaP shot, immunohistochemistry, and next‐generation sequencing (NGS). By evaluating the sensitivity, specificity, and feasibility of these technologies, NGS was shown to be the first choice for detecting very‐low‐grade somatic mosaicism (Braunholz et al., 2015; Ihle et al., 2014; Miyatake et al., 2014). However, there is no data as a reference for suitable read coverage for different ratios of mosaicism detection. Last, the assessment of unknown mosaicism events without specific mutation indications in clinical diagnostics is definitely much more challenging. There is no system strategy for evaluating germline mosaicism, which hinders the management of mosaicism for geneticists.

Here, we present our study of a Chinese family with one child affected by Alport syndrome (AS). AS is a genetically heterogeneous disorder characterized by hematuria, progressive renal failure, hearing loss, and ocular abnormalities (Gubler et al., 1981). Approximately 85% of AS (MIM: 301050) cases are X‐linked with the *COL4A5* (Xq22.3(MIM: 303630)) gene, and approximately 15% are autosomal recessive AS (MIM: 203780) and associated with *COL4A3* (2q36.3(MIM: 120070)) and *COL4A4* (2q36.3(MIM:120131)) (Barker et al., 1990; Feingold et al., 1985; Mochizuki et al., 1994). Autosomal dominant inheritance AS (MIM: 104,200) is rare (van der Loop et al., 2000). Recently, 2.2% (4/186)‐10.7% (3/28) somatic mosaicism of *COL4A5* was reported (Fu et al., 2016; Plant, Boye, Green, Vetrie, & Flinter, 2000), and maternal gonadal mosaicism of *COL4A4* and *COL4A5* was also suspected in recent studies (Anazi et al., 2014; Okamoto, Nozu, Iijima, & Ariga, 2019). In the present family, only one affected boy was found to inherit a familial *COL4A3* mutation from the mother and a germinal mosaic mutation from the father, which was found using combined MicroSeq and targeted next‐generation sequencing (TNGS) on semen. In addition, we performed TNGS on prepared chimeric mutant samples of different proportions and revealed the detection sensitivity for different mosaic proportions at different read coverages. Through this case, we tried to solve three problems: (1) Identification of an allele source of a de novo mutation in a single patient. (2) Assessment of the mosaic mutation ratio using TNGS. (3) Establishment of detection tactics for germline mosaicism with a single patient.

## 2 MATERIALS AND METHODS

### 2.1 Ethical compliance

The parents and both children were enrolled after they signed written informed consent forms, and the study was approved by the Ethics Committee of the Reproductive Genetic Hospital of CITIC‐Xiangya (LL‐SC‐2019‐028).

### 2.2 DNA preparation

Blood was drawn in EDTA tubes for DNA extraction and in heparin tubes for chromosome preparation. Semen samples were collected from the father.

Genomic DNA from peripheral blood samples was extracted using a QIAamp DNA Mini Kit (Qiagen). A spectrophotometer (NanoDrop) was used to determine the purity and concentration of the gDNA in the sample.

Genomic DNA of sperm samples was obtained from 1ml semen, which was centrifuged to obtain the sperm pellet and remove the supernatant. Sperm pellets were lysed with 20 µl 20 mg/ml proteinase K and 1 MDTT; then, 400 µl of digestion buffer (10 mM Tris‐Cl pH 8.0, 1 mM EDTA, 0.1% SDS, 0.5% N‐lauryl sarcosine) was added at 56°C for 40 min. DNA was precipitated in three volumes of 100% ethanol and dissolved in TE buffer.

### 2.3 Whole‐exome sequencing

Exome capture was performed using the TruSeq Exome Enrichment Kit (Illumina) following the manufacturer's protocol. Samples were prepared as an Illumina sequencing library, and in the second step, the sequencing libraries were enriched for the desired target using the Illumina Exome Enrichment protocol. The captured libraries were sequenced using an Illumina HiSeq 2000 Sequencer. The reads were mapped against UCSC hg38 (http://genome.ucsc.edu/) by BWA (http://bio‐bwa.sourceforge.net/).

### 2.4 Sanger sequencing

For candidate variant validation and pedigree analysis, specific PCR primers were designed for the region of the mutations in *COL4A3* (NM 000091.4): *COL4A3*‐22F: 5'‐CCATGCTTTCTCAGTTGCAGAT‐3' and *COL4A3*‐22R: 5'‐ TTGGGATCATTGTTATCTCAGGGG‐3'; *COL4A3*‐35F: 5'‐ATTCGCAGGAAATCCAGGTGAG‐3' and *COL4A3*‐35R: 5'‐ACACGCCAACTATCAACACC‐3'. Bidirectional sequencing was performed using an ABI 3730‐automated sequencer (Applied Biosystems).

### 2.5 Paternity test

Sixteen different human polymorphic markers (D8S1179, D21S11, D7S820, CSF1PO, D3S1358, TH01, D13S317, D16S539, D2S1338, D19S433, vWA, TPOX, D18S51, Amel, D5S818, and FGA) were genotyped to confirm the paternity of the parent and the proband.

### 2.6 MicroSeq

**Figure 1:**
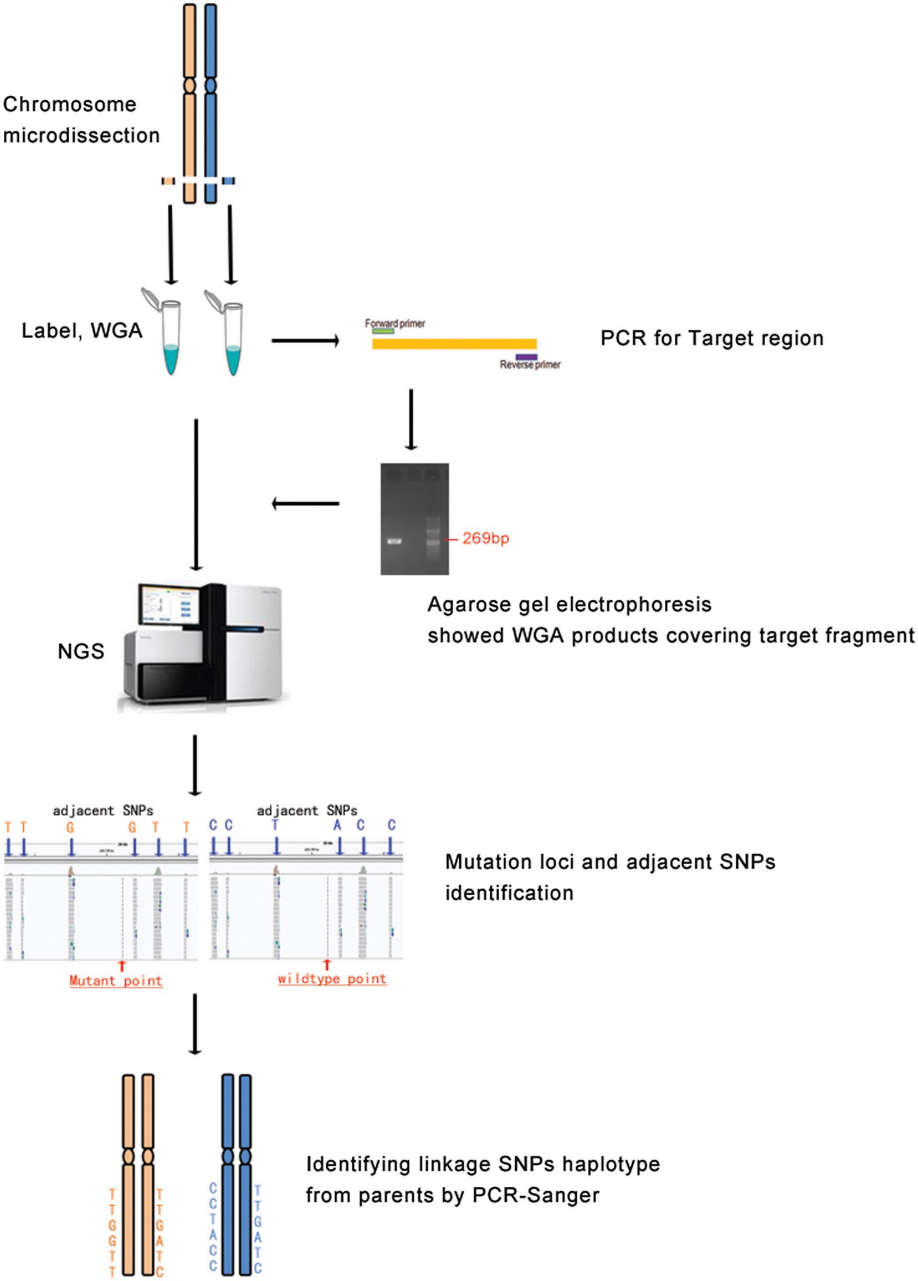
Schematic diagram of haplotype identification for de novo mutations. The targeted region of *COL4A3* on the pair of homologous chromosomes was microdissected and amplified via WGA. The amplified products were first confirmed to cover the mutant locus fragment and then sequenced using NGS. The precise mutation point and the adjacent SNPs were determined. Informative linkage SNPs were confirmed by PCR‐Sanger sequencing in the parents. Thus, the parent chromosome source of the mutant site was determined

To simultaneously determine whether the candidate mutations are *in cis* or *in trans* and the chromosome origin of the de novo mutation, MicroSeq was performed as shown in Figure 1, and the entire process was divided into three parts.

#### 2.6.1 Chromosome microdissection

The target region of the homologous pair of chromosome 2 was initially dissected under inverted microscopy. The procedure was improved by referring to the methods described in our previous literature (10). In short, eight copies of the region in 2q36 covering the mutation were dissected from G‐banding metaphase spreads of the patients' peripheral blood samples using glass needles. Then, the chromosome fragment was placed in 9 µl of collection fluid in EP tubes, and each whole‐genome amplification (WGA) was performed using a WGA4‐GenomePlex Single Cell Whole Genome Amplification Kit (Sigma‐Aldrich) following the manufacturer's protocol. The WGA products, which were verified covering the mutation sites by PCR using specific primers, were subjected to NGS testing.

#### 2.6.2 NGS of WGA product

The sequencing library was prepared by an Ion Xpress Library Kit from Life TechCompany according to the instruction procedures. Library DNA was diluted to 2.5 pg/µl, amplified by a Ion PGMTM Template OT2 200 Kit, run for 5.5 hr and sequenced on a PGM (Ion PGM Sequencing 200 Kit v2) plate with a 318 chip; the number of sequencing flows was set to 460, the reference chromosome was hg38, the Ion Xpress barcode was 1, and the reaction was run for 4.5 hr.

#### 2.6.3 Haplotype analyses

Using self‐designed software, the target mutation point was found, and all of the SNP loci in the range of 0–5 Mb up and downstream of the mutation site were listed. According to the population heterozygosity of the SNP, six loci were selected for the following haplotype analysis. Sanger sequencing was conducted toward the selected SNPs on the parents and proband with peripheral blood DNA. By linkage genetic analysis with informative SNPs, the parent source of the target mutation was determined.

### 2.7 TNGS analysis

Spermatozoa DNA and peripheral blood DNA of the father was sequenced with TNGS in the mutant region. The DNA samples were first amplified using PCR with specified primers for the target mutation. Then, purified PCR products were amplified using Tag ReadyMix (TaKaRa) and prepared as a sequencing library with the HTP Library Preparation Kit following the manufacturer's protocol. The libraries were finally sequenced using an Illumina NextSeq 500 Sequencer. The reads were mapped against the human hg38 database.

To determine the detection sensitivity for different proportions of chimeras at different depths with NGS, mosaic mutant samples of different proportions of 3% and 0.1% were prepared by mixing blood DNA samples with heterozygous mutations and wild‐type DNA samples at known sites. Then, mosaic samples were analyzed using TNGS as described above.

## 3 RESULTS

### 3.1 Clinical report

**Figure 2:**
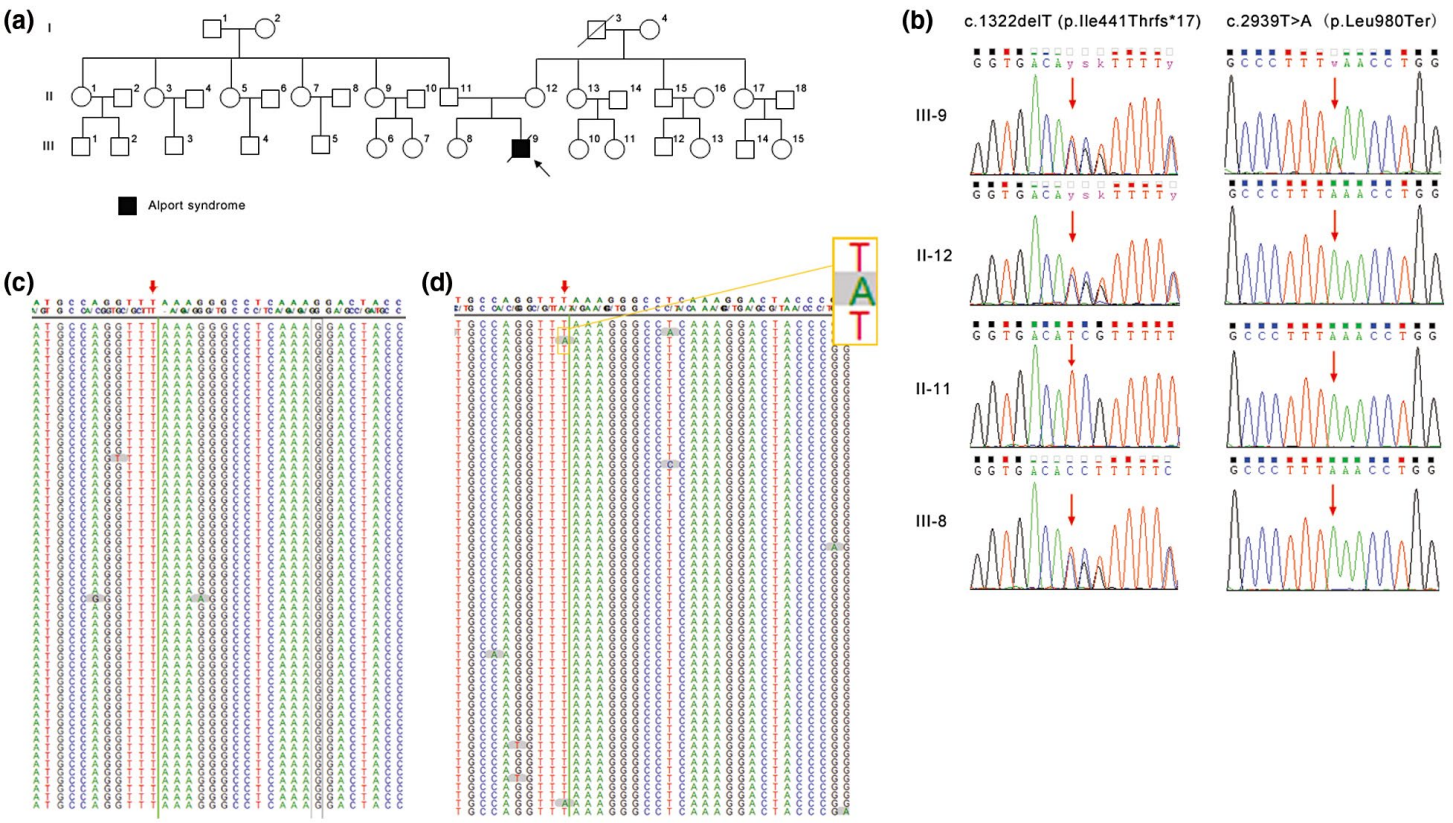
Pedigree of the investigated family and their sequences of *COL4A3*. (a) Family pedigree. The proband is shown as a black square with an arrow. (b) Sanger sequencing of DNA from peripheral blood on sites c.1322T and c.2939T of *COL4A3*. C,D. TNGS analysis of site c.2939T of *COL4A3* in a peripheral blood DNA sample (c) and a sperm DNA sample (d) from the father. No mutant nucleotide was detected from the blood DNA sample, but a low percentage of mutant adenine was detected from the sperm DNA sample

The propositus, a 13‐year‐old boy from the Chinese family of the Han ethnic group, is the second child of the healthy nonconsanguineous couple (Figure 2a). At 3 years of age, the propositus had microhematuria (microscopic erythrocyte:++) and moderate proteinuria at 5.0 g/L. Kidney biopsy was taken and pathologically diagnosed with mild mesangial proliferative glomerulonephritis under electron microscopy and with focal segmental glomerulosclerosis under light microscopy. Then, he had progressive deterioration of renal function. The patient was diagnosed with chronic renal insufficiency with uremia at age 12. In the same year, he was found to have renal anemia, renal hypertension, binocular retinopathy, and a macular hole in the right eye. Based on these clinical findings, a clinical diagnosis of AS was determined. His parents came to our hospital for genetic counseling. A year later, the patient died of hypertensive encephalopathy.

### 3.2 Mutation identification and detection of a suspected de novo mutation

Using exome sequencing, two heterozygous variants, c.1322delT (p.Ile441Thrfs*17) and c.2939T>A (p.Leu980Ter), in the *COL4A3*gene were discovered from the propositus, both of which are novel variants that have not been reported in any public databases or literature. c.1322delT was a frameshift mutation and may cause the encoded residues to change from position 441 and generate termination 17 codons from that point. Mutation c.2939T>A was a nonsense mutation and may result in a premature stop codon at residue 980. Both mutations would result in a truncated protein and are classified as “LikelyPathogenic” based on the American College of Medical Genetics and Genomics (ACMG) interpretation of variants (Richards et al., 2015).

Pedigree analysis by Sanger sequencing toward these two mutations showed that the mother and the older daughter were heterozygous for c.1322delT (p.Ile441Thrfs*17). The other mutation was absent from the blood lymphocytes of the parents and the older sister (Figure 2b). Then, a paternity test confirmed the paternity of the parents and the proband. To further determine whether the father has somatic mosaicism, TNGS was performed on blood lymphocyte DNA from the father, and c.2939T>A (p.Leu980Ter) was confirmed to be absent from a blood sample from the father at a depth coverage of 10,000X (Figure 2c).

### 3.3 Paternal chromosome origin determination of the de novo mutation

**Figure 3:**
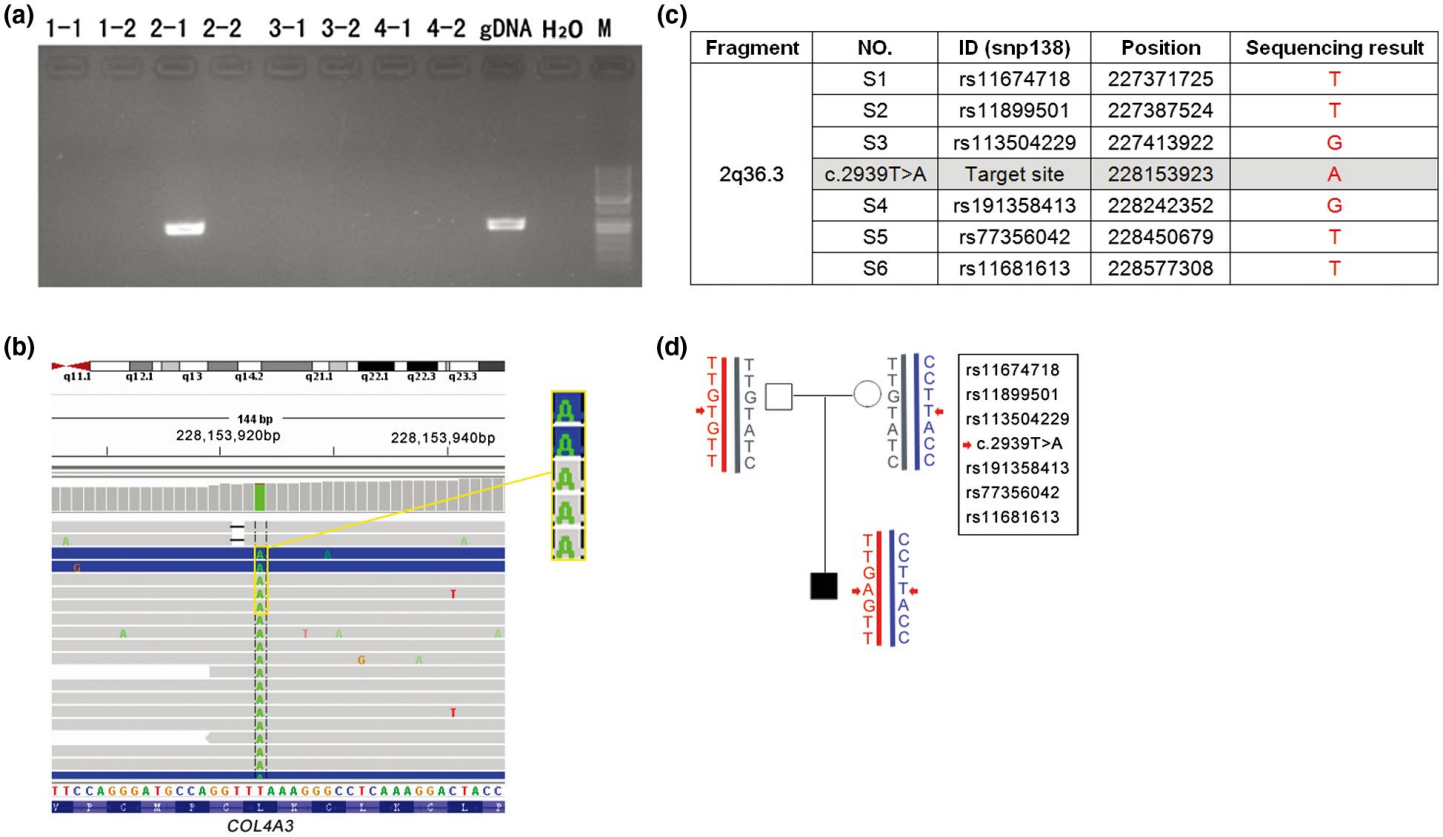
Results of MicroSeq for the propositus and SNP haplotypes. (a) Electrophoretogram of amplification of the mutation site using PCR for microdissection pieces of WGA products. “1‐1”, “1‐2”, “2‐1”,“2‐2”, “3‐1”,“3‐2”, “4‐1”, and “4‐2” indicate each allele of a chromosome region of 2q36 from four metaphase chromosome slides. “gDNA” was a positive control, and “H_2_O” was a negative control; “M” was a DNA marker. The results showed that only one tube of WGA product from a single allele of slide 2 covered the target fragment. (b) Mutant A was detected by TNGS from the “2‐1” WGA product. (c) The sequencing result of site c.2939 and the adjacent six SNPs in the “2‐1” chromosome fragment. The index genotype of the SNPs for the mutant allele was “TTGGTT”. (d) Sanger sequencing of the six SNPs and mutant site for the proband and the parents. Position c.2939 is marked with an arrow. Based on the six SNPs with the sequence “TTGGTT”, the mutant c.2939T>A allele was inherited from the father

As shown in Figure 3, the mutant type c.2939A was detected to be inherited from the paternal allele by MicroSeq; in other words, c.2939T>A (p.Leu980Ter) was *in trans* with c.1322delT (p.Ile441Thrfs*17) since the latter mutation was demonstrated to be inherited from the mother.

### 3.4 Paternal germline mosaicism identification

We performed TNGS on DNA from the father's sperm and found that the percentage of c.2939A was 2.65% (Figure 2d, Table 1) at a 10,000X read depth. In addition, the mutation frequency calculated at different read depths was basically equal, ranging from 2.24% to 2.86%. Thus far, the paternal germline mosaicism origin for the “de novo” mutation was clear. Since the mutant fraction in sperm was less than 3%, the recurrence risk of the next child was estimated to be lower than 1.5%. Considering the great economic and psychological pressure brought by this disease in their family, this couple choose preimplantation genetic diagnosis (PGD) to have a third child free of both mutations.

**Table 1:** Sequencing results of TNGS at different read coverages for peripheral blood samples, sperm from the father, and self‐prepared mosaic mutation samples of 3% and 0.1%

Sequencing depth (X)	Mosaic fraction			
	Peripheral blood	Sperm	3%	0.1%
200	0	0	3%	0
500	0	2.86%	3%	0
1,000	0	2.75%	3.40%	0
2,000	0	2.71%	3.30%	0
5,000	0	2.24%	3.72%	0
10,000	0	2.65%	3.45%	0

### 3.5 Detection sensitivity for different proportions of chimeras with TNGS

The results of DNA analysis of semen by TNGS provide conclusive evidence of the paternal origin of the mutation and help determine the proportion of mutant sperm. However, no reference data are available for the detection sensitivity of mosaicism using TNGS at present. Here, we carried out TNGS on self‐prepared mosaic samples of 3% and 0.1% allele frequencies at the same site c.2939T>A and screened for the mutant allele frequencies at different read depths. We found that the allele frequency calculated from reads was basically equal to the actual mosaic frequency. The results showed that 200X depth succeeded in revealing a mosaic mutation of 3% but did not work for less than 3% in the sperm sample. Detection efficiencies in 500X depth coverage were stable for sperm DNA and self‐prepared samples. In addition, when the mosaic fraction was down to 0.1%, the mutation could not be detected using TNGS, even at a 10,000X depth coverage. The detailed results are shown in Table 1.

## 4 DISCUSSION

**Figure 4:**
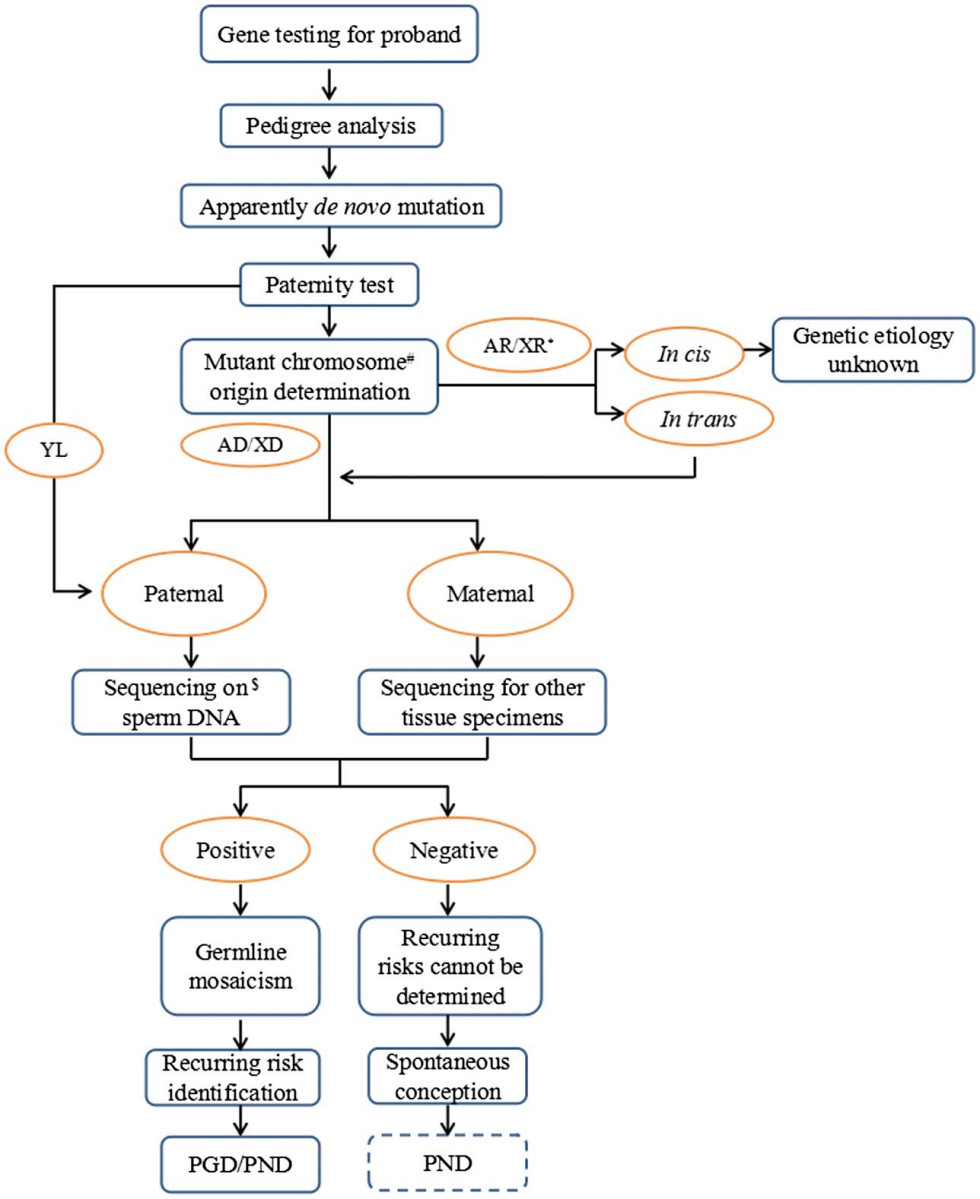
Scheme for the diagnosis of germline mosaicism for an apparent de novo mutation in a single patient. AD, Autosomal dominant inheritance; AR, autosomal recessive inheritance; PGD, Preimplantation genetic diagnosis; PND, prenatal genetic diagnosis; XD, X‐linked dominant inheritance; XR, X‐linked recessive inheritance; YL, Y‐linked inheritance. ^#^When a de novo mutation is identified based on regular genetic testing, MicroSeq might be the only method for mutant chromosome origin determination in a single patient. *For diseases with autosomal recessive or X‐linked recessive inheritance, mutant chromosome origin analysis is essential for genetic diagnosis. The existing mutations could be considered associated with the disease only when the mutations were proven to be *in trans* with each other after chromosome origin analysis.^$^TNGS is a recommended method for mosaic mutation testing on DNA specimens from sperm or other tissues

In this case, regardless of the methodology used, compound heterozygosity was eventually identified for two novel mutations in the *COL4A3* gene, and germline mosaicism was identified as a source of the paternal allele for the apparent de novo mutation. We established a pipeline for detecting germline mosaicism in a single patient, as shown in Figure 4, since germline mosaicism would be considered when multiple patients are present. Strategies may vary based on clinical presentation and availability of testing, and the main principles should be generalizable.

Once a de novo mutation was suspected, to avoid excess labor and economic waste caused by specimen error, the paternity test was a necessary step before the following work. In the present case, after the paternity was confirmed, additional TNGS on peripheral blood DNA was performed for the exclusion of paternal somatic mosaicism since the Sanger sequencing used for routine pedigree analysis is not sensitive to mosaicism detection; in addition, it was highly suspected that the paternal germline was involved in the existing results.

In our study, mutant chromosome source determination was performed using MicroSeq since the traditional method of haplotype analysis using microsatellite markers was not applicable because there are not enough informative samples in the index family. To the best of our knowledge, this is the first application of MicroSeq in the clinical molecular diagnosis of diseases of Mendelian inheritance.

**Figure 5:**
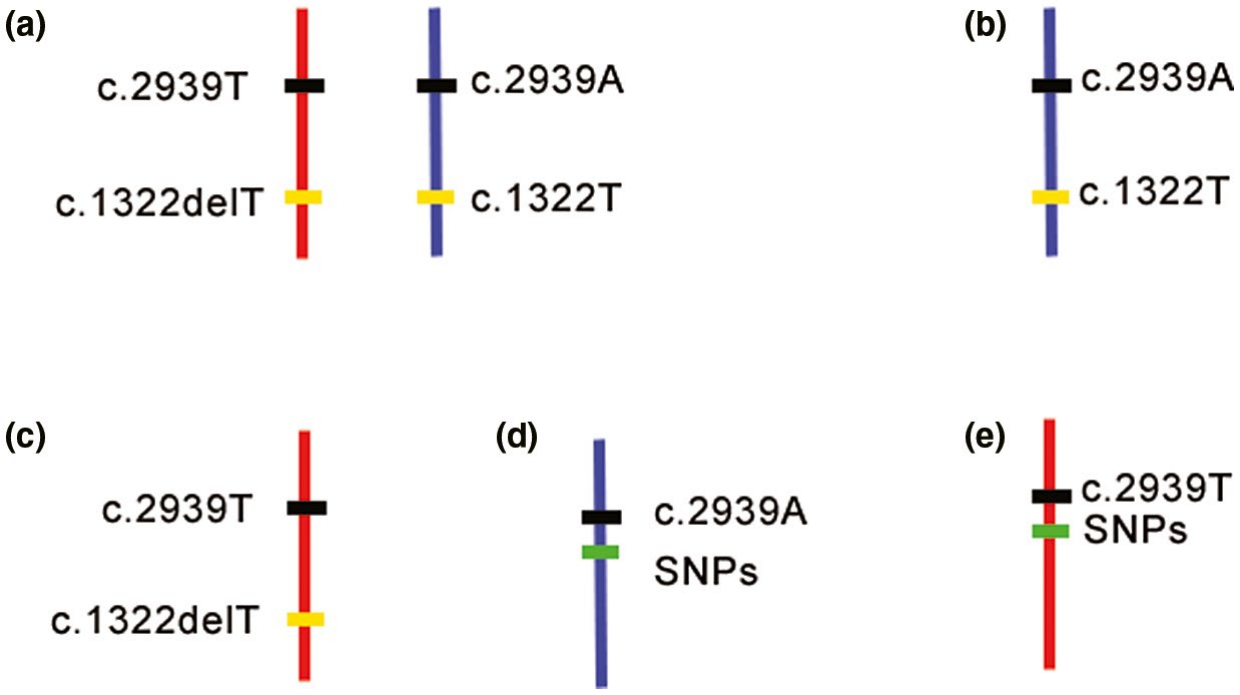
Five possible scenarios for sequencing the CM fragments in the present case. (a) The best result for which wild‐type and mutant sites for both mutations (c.2939T>A and c.1322delT) in homologous chromosomes were detected. (b, c) Only one allele covering the two mutations was detected. Either the mutant base of the de novo mutation and the wild‐type base of the known mutation (d) or the opposite combination (c) was informative. (d) The mutant base of the de novo mutation and the linkage SNPs were detected as in the present case. (e) The wild‐type base of the de novo mutation and the linkage SNPs were detected. The mutant base can be inferred to be on the other homologous chromosome from this result. However, we recommend continuing the experiments until any one of the first four results occurs. Since this result does not directly show the relationship between the target mutation and the known mutation nor the chromosome on which the mutant base of the de novo mutation is located, chromosomal recombination cannot be excluded based on these results

In this study, using the MicroSeq approach, we were able to precisely map the mutant allele in the patient sample independently and clarify the chromosomal origin simultaneously. This approach combines CM and NGS techniques. Precise dissection of chromatin covering the target point minimizes the following NGS cost. NGS of amplified dissected DNA can reliably and accurately obtain the sequence of the target fragments, and this ability helps to precisely identify the target sites as well as nearby SNPs. It takes only 5 days for CM, and a regular library construction protocol is suitable for the following NGS (Hu et al., 2016). Because this is the first use of MicroSeq in mutation detection, the efficiency of the amplification of the excised fragments needs to be optimized. The mutant site was captured in only one of the eight fragments from four cells. Theoretically, when sequencing the CM fragments, the ideal situation would be to detect both alleles for c.2939T>A and c.1322delT in homologous chromosomes, as shown in Figure 5a. However, limited by the efficiency of the WGA kit, another four scenarios might occur (Figure 5b–e). In our case, we obtained the sequence of the mutant base of the “de novo” variant c.2939A and the adjacent SNPs, as shown in Figure 5d. c.2939A was detected in one of the chromosome 2q36 fragments from the proband (Figure 3a,b) by MicroSeq, and six informative linkage SNPs (rs11674718, rs11899501, rs113504229, rs191358413, rs77356042, and rs11681613) were selected from the sequencing results; the corresponding SNP genotype was “TTGGTT” (Figure 3c). Then, six SNPs were used to perform Sanger sequencing in the proband and parents for haplotype analysis. The results revealed that the c.2939T>A mutation was inherited from the paternal chromosome (Figure 3d), which also proved that the two mutations were *in trans* with each other in the proband. Thus far, we can conclude that the patient's phenotype was very likely the result of a complex heterozygous mutation of *COL4A3* and that the “de novo” mutation was located on the paternal chromosome.

Then, TNGS for sperm DNA confirmed the paternal germline mosaicism and showed the mosaic fraction. In this investigation, the mosaic proportion detected in sperm DNA was basically equal at different read depths. A mosaic ratio of 2.86% was detected. This is close to that of previous research showing that TNGS was able to detect somatic BRAF mutations with an allele frequency as low as 2% (Ihle et al., 2014). Although the experimental data are limited, the existing results suggest that 500X depth coverage might be sufficient to serve the detection needs for mosaic mutations with a low allele frequency. Therefore, to save costs, we believe that read coverage at a depth of 500X may be worthwhile for conventional NGS, and even many mosaic mutations are often regarded as background noise and missed in routine genetic testing. Reanalysis of the low‐frequency mutations in the original reads will provide an opportunity to diagnose mosaicism with this read coverage. In addition, the mosaic ratio calculated from the NGS reads was basically equal to the actual proportion from the results of the self‐prepared mosaic samples. In germinal mosaicisms, the heritability of any disease‐causing mutation depends on its percentage of positive gonadal cells, indicating that it was clinically relevant to calculate the mutation allele frequency based on the NGS reads and to infer the probability of inheritance.

We can say the two results from MicroSeq and semen sequencing are complementary and independent in the present case. When the semen sample is not available or when the de novo mutation is inherited from the maternal allele, the molecular haplotype of MicroSeq is essential for the final genetic diagnosis for recessive inherited diseases. The present case addressed haplotype identification in a single patient and provided a solution for determining paternal germline mosaicism. Determining maternal germ cell mosaicism is limited by the difficulty of oocyte acquisition, and circumstantial evidence from multiple sources of different germinal layers (Campbell, Shaw, Stankiewicz, & Lupski, 2015) can be used as speculative results and merits consideration.

In conclusion, the results of our study clarified a paternal germline mosaicism for the de novo mutation c.2939T>A (p.Leu980Ter) and made PGD and prenatal diagnostics available for the couple. Technically, although the experimental conditions need to be optimized, MicroSeq is still a valuable tool in molecular haplotype determination in a single‐affected individual and plays a key role in chromosome tracing for de novo mutations and determining germline mosaicism in clinical genetic detection. TNGS can be used to assess the mosaic ratios of known sites. For NGS, the 500X depth could be set as the read coverage and may serve the detection needs for both routine gene detection and mosaic mutation detection of alleles with low frequency. Furthermore, we think that the workflow of our study is worthy to be used in routine clinical applications to rule out germinal mosaicism when a suspected de novo mutation occurs. To avoid potential recurrence risk, a de novo mutation could be identified only after germline mosaicism was excluded when there is only a single‐affected individual with genetic predisposition to a particular disease.

## CONFLICT OF INTERESTS

The authors declare no conflicts of interest.

## AUTHORS CONTRIBUTIONS

Q‐JZ designed the research, validated the data, and revised the manuscript; G‐XL recruited patients, performed the clinical evaluation and supervised the study; C‐LD carried out genomic analysis and wrote the paper. D‐HC handled Microseq. W‐NL performed genetic analysis on sperm. S‐CZ Managed ethics approval and sample collection.

## Data Availability

The data that support the findings of this study are available from the corresponding author upon reasonable request.
